# Association of Body Mass Index (BMI) with Lip Morphology Characteristics: A Cross-Sectional Study Based on Chinese Population

**DOI:** 10.3390/diagnostics13050997

**Published:** 2023-03-06

**Authors:** Yiyin Chen, Hongmei Yang, Zhijin Zheng, Xiaoqi Zhang, Xinyu Yan, Hu Long, Wenli Lai

**Affiliations:** 1 State Key Laboratory of Oral Diseases, National Clinical Research Center of Oral Diseases, Department of Orthodontics, West China Hospital of Stomatology, Sichuan University, No. 14, Section 3, Ren Min South Road, Chengdu 610041, China; 2 West China Hospital of Stomatology, Sichuan University, No. 14, Section 3, Ren Min South Road, Chengdu 610041, China

**Keywords:** lip morphology, body mass index, BMI, cephalometric, multivariable regression

## Abstract

Background: Lip morphology is essential in diagnosis and treatment of orthodontics and orthognathic surgery to ensure facial aesthetics. Body mass index (BMI) has proved to have influence on facial soft tissue thickness, but its relationship with lip morphology is unclear. This study aimed to evaluate the association between BMI and lip morphology characteristics (LMCs) and thus provide information for personalized treatment. Methods: A cross-sectional study consisted of 1185 patients from 1 January 2010 to 31 December 2020 was conducted. Confounders of demography, dental features, skeletal parameters and LMCs were adjusted by multivariable linear regression to identify the association between BMI and LMCs. Group differences were evaluated with two-samples *t*-test and one-way ANOVA test. Mediation analysis was used for indirect effects assessment. Results: After adjusting for confounders, BMI is independently associated with upper lip length (0.039, [0.002–0.075]), soft pogonion thickness (0.120, [0.073–0.168]), inferior sulcus depth (0.040, [0.018–0.063]), lower lip length (0.208, [0.139–0.276]), and curve fitting revealed non-linearity to BMI in obese patients. Mediation analysis found BMI was associated with superior sulcus depth and basic upper lip thickness through upper lip length. Conclusions: BMI is positively associated with LMCs, except for nasolabial angle as negatively, while obese patients reverse or weaken these associations.

## 1. Introduction

Soft tissue aesthetics, as the major motivation of patients seeking orthodontic and orthognathic treatment, has become an important concern in orthodontic treatment planning [1,2,3]. Therefore, it is of great value to explore the potential influencing factors related to soft tissue aesthetics and to make personalized treatment plans for patients. Recent studies have paid increasing attention to lip profile, as it has been proved to be a key feature affecting facial esthetic perception [4]. However, increasing evidence has shown that, in addition to hard tissue morphology, soft tissue morphology is affected by many factors, including heredity and environment (race, age, gender, etc.). Specifically, compared with females, males were found to have more prominent and thicker lips [5]. Besides, dental features such as dental crowding, occlusal relationship, and especially the incisor position, also have an impact, with the anterior and posterior position of the upper incisor proving to be closely related to upper lip thickness [6]. Moreover, recent study has demonstrated that the upper lip morphology varies significantly between different skeletal patterns [7].

Body Mass Index (BMI), a commonly used value to measure the body shape and health status, is calculated as weight in kg divided by height in m squared [8]. Previous studies have found that BMI is associated with various systemic diseases [9]. Concerning oral health, some evidence exists that there might be an association between increased BMI and an increased risk for caries [10,11], periodontal diseases [12], root dilaceration [13] and less cooperation [14]. In addition, it has been proved that obesity can affect facial bone and soft tissue structures by affecting growth and development, bone metabolism and fat distribution [15]. For example, mandibular growth and lower facial height were found to be significantly associated with BMI [16]. Overweight subjects were found to have larger maxillary width and obese people were found to have larger maxillary length and mandibular width [17]. Besides, many studies have determined that BMI is one of the key factors affecting facial soft tissue thickness (FSTT) [18,19], which increased with increase in BMI. Specifically, overweight subjects were found to have thicker nasion soft tissue, whereas obese subjects were found to have thicker pogonion, glabella and gnathion soft tissue. In recent years, research on lip aesthetics has been carried out worldwide and standard values have been established in some populations [20,21]. Previous studies have mainly explored facial soft tissue characteristics based on age, gender, race, and skeletal patterns. Recent stereophotogrammetric analysis first reported the association between larger BMI and increased linear lip measurements, which may suggest that increases in BMI were associated with directional lip stretch [22].

Current studies on the relationship between BMI and facial soft tissue mainly focus on its thickness (FSTT). There are few studies concerning lip morphology, and the relationship between BMI and lip morphology remains unclear. In addition, many existing studies have defects such as limited sample size or imperfect statistical methods. Given the nonnegligible influence of BMI on facial soft tissue and bone structures, and the importance of lip morphology in aesthetic considerations for orthodontic treatment, it is necessary to explore the clear association between BMI and lip morphology, to provide a basis for more accurate diagnosis and to help orthodontists develop more personalized aesthetic treatment plans for patients with different BMI. Therefore, a statistically well-designed study with a larger sample size has been conducted in a Chinese population, aimed to (a) explore the average value of lip morphology characteristics and reference value for BMI in Chinese population; (b) compare lip morphology characteristics among four BMI categories; (c) to explore the lip characteristics which are independently affected by BMI by adjusting for various confounding variables; and (d) to explore the mediators between BMI and lip characteristics. The null hypothesis of this study was that lip morphology characteristics did not differ significantly between four BMI categories.

## 2. Materials and Methods

### 2.1. Study Population and Data Collection

**Figure 1:**
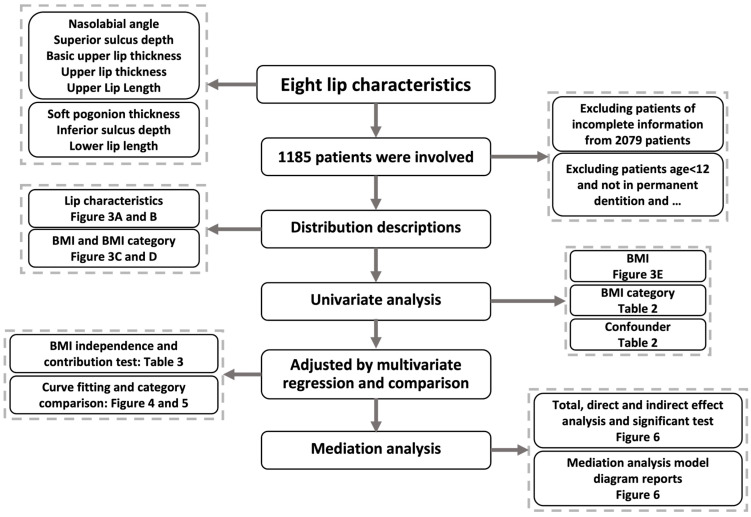
Study flow chart demonstrates the analysis process and content of the research.

The study was a cross-sectional study, which was reported following the Strengthening the Reporting of Observational Studies in Epidemiology (STROBE) guideline [23]. Figure 1 shows the flow chart of the analysis process and research contents. Patients who received consecutive orthodontic treatment in the Department of Orthodontics, West China Hospital of Stomatology, Chengdu, Sichuan, from 1 January 2010 to 31 December 2020, were identified retrospectively. The exclusion criteria were: (a) participants aged < 12 y; (b) participants with a history of orthodontic treatment; (c) participants who do not have permanent dentition; (d) participants without the required complete baseline information. Prior to orthodontic treatment, all participants received a series of examinations, including demographic questionnaires, intraoral and facial photographs, plaster, digital dental models and radiographic examinations. The above data were collected for subsequent analysis, and informed consent was obtained from all adult participants and the guardian of each minor.

**Figure 2:**
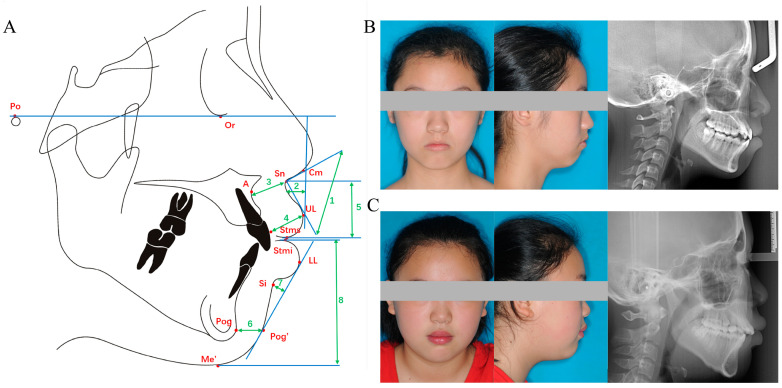
Schematic diagrams of the eight lip characteristics used in the present study and representative patients’ profiles. (**A**) The description of eight lip characteristics. 1: Nasolabial angle, 2: Superior sulcus depth, 3: Basic upper lip thickness, 4: Upper lip thickness, 5: Upper lip length, 6: Soft pogonion thickness, 7: Inferior sulcus depth, 8: Lower lip length. (**B**) A representative underweight patient profile. (**C**) A representative overweight patient profile.

Lateral cephalometric radiographs were taken using the same device (Veraviewepocs, Morita, Kyoto, Japan). Patients were in their natural head position and centric occlusion, and were instructed to stay relaxed and not compress their lips during exposure. Pre-treatment cephalometric radiographs were imported into Dolphin imaging software version 11.9.07.23 (Patterson Dental, Los Angeles, CA, USA), independently traced and measured by two experienced orthodontists, the mean values of which were used for subsequent analyses. A total of 7 linear parameters and one angular parameter were measured using cephalometric landmarks. Landmarks, reference lines and measurements used in this study are shown in Figure 2A. The cephalometric landmarks are defined in Table 1.

### 2.2. Lip Characteristics and BMI

Lip morphology characteristics (LMCs) were described using eight indices: nasolabial angle (NLA), superior sulcus depth (SSD), basic upper lip thickness (BULT), upper lip thickness (ULT), upper lip length (ULL), soft pogonion thickness (SPT), inferior sulcus depth (ISD) and lower lip length (LLL). Specifically, (a) NLA is the intersection angle between the line Cm-Sn and the line Sn-UL; (b) SSD is the distance from the most concave point of upper lip to the line perpendicular to the Frankfort (FH) plane (the line Or-Po); (c) BULT is the distance between point Sn and the point 3 mm below point A; (d) ULT is the distance from point UL to the labial surface of the upper central incisor; (e) ULL is the distance between two parallel lines in the FH plane through point Sn and point Stms; (f) SPT is the distance between point Pog and Pog’; (g) ISD is the vertical distance from point Si to the line LL-Pog’; (h) LLL is the distance between two parallel lines in the FH plane through point Stmi and point Me’.

BMI was calculated using objectively measured height and weight records from demographic questionnaires prior to orthodontic treatment. According to the World Health Organization standards, BMI can be divided into the following four categories: underweight (BMI < 18.5 kg/m^2^); normal weight (18.5 ≤ BMI < 25.0 kg/m^2^); overweight (25.0 ≤ BMI < 30 kg/m^2^) and general obesity (BMI ≥ 30 kg/m^2^). Figure 2B shows a representative underweight patient profile and Figure 2C shows a representative overweight patient profile.

### 2.3. Covariates

Demographic information on age and gender was obtained from the medical record system of the hospital. Dental features including crowding, molar relationship, overbite, and overjet were assessed based on intraoral photographs, dental models, and medical examination records at the first visit. Molar relationship is diagnosed as class I when the mesi-obuccal cusp of the upper first molar (U6) occludes with the buccal groove of the lower first molar (L6), as class II-1 when U6 is mesial to L6 and upper incisors proclined and as class II-2 when upper incisors retroclined (U1-SN < 100°), and as class III when U6 is distal to L6. In addition, crowding evaluated both in upper and lower dentition is defined as I (<4 mm), II (4~8 mm), and III (≥8 mm). Cephalometric indices on skeletal and incisor parameters were obtained, mainly including the development and relative position of the jaw (SNA, SNB, ANB, SN-MP, FH-MP), as well as the inclination and position of the central incisor (U1-NA, U1-SN, L1-NB, L1-MP). To control for potentially confounding effects and make the results convincing, the above variables were adjusted for covariates in our study, according to previously literature [7], which apparently have an effect on the appearance characteristics of soft tissues. Besides, the eight LMCs were also considered as confounders because they may influence each other.

### 2.4. Statistical Analysis

Categorical variables were expressed as frequencies (percentages) and continuous variables were described as means (standard deviations, SDs) or medians (interquartile ranges, IQRs). Demographic and clinical characteristics were presented according to BMI categories and compared by one-way ANOVA test and Chi-square test as appropriate. Spearman correlation analysis was used to investigate the relationships of eight LCMs to each other. Multivariable linear regression models were used to explore the association between BMI with LCMs when considering confounders. Among this process, four models were adjusted by confounders: model 1 for basic diagnosis information (“Age”, “Gender”, “Molar Relationship”, “Upper crowding”, “Lower crowding”, “Overbite” and “Overjet”), model 2 for anterior teeth and skeletal information (“SNA”, “SNB”, “ANB”, “SN-MP”, “FH-MP”, “U1-NA (mm)”, “U1-SN” “L1-NB (mm)” and “L1-MP”), model 3 for LMCs themselves (NLA, SSD, BULT, ULT, ULL, SPT, ISD and LLL), and model 4 for all of these. Adjusted LCMs value were compared among four BMI categories by two-samples *t*-test and one-way ANOVA test. Loess (Local Polynomial Regression Fitting) was employed to describe the variation tendency of eight adjusted LCMs with the BMI. The multi-variable linear regression results were expressed as coefficient and 95% confidence interval (CI). Regression-based mediation analysis was used to distinguish the direct effect of BMI on LMCs, and the indirect effect mediated by ULL. Three estimates were obtained as follows: (a) total effect, i.e., the overall association between BMI and LMCs, including direct and indirect effects; (b) direct effect, the association between BMI and LMCs; and (c) indirect effect, the association between BMI and LMCs, mediated by ULL. All statistical analyses and results’ visualization were performed using R software (version 4.1.2). *p*-value < 0.05 was considered statistically significant.

## 3. Results

### 3.1. Study Participant Characteristics

**Figure 3:**
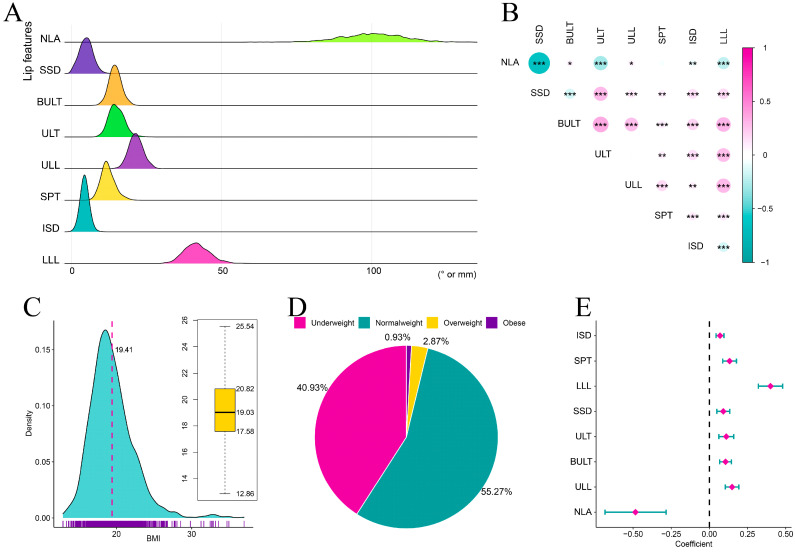
Data description of distributions for lip characteristics and BMI. (**A**) Data distributions of eight lip characteristics. (**B**) Correlations among the eight lip characteristics. (**C**) Data distribution of BMI characteristics. (**D**) Data distribution of four BMI categories. (**E**) Univariate analysis comparing BMI and eight lip characteristics and results demonstrated by forest-plot. NLA: Nasolabial angle, SSD: Superior sulcus depth, BULT: Basic upper lip thickness, ULT: Upper lip thickness, ULL: Upper lip length, SPT: Soft pogonion thickness, ISD: Inferior sulcus depth, LLL: Lower lip length, BMI: Body mass index. *: *p* < 0.05, **: *p* < 0.01, ***: *p* < 0.001.

A total of 2079 patients were initially identified, with 1185 remaining as final participants after applying exclusion criteria. Among the 1185 including participants, the eight LMCs were normally distributed according to Kolmogorov-Smirnov tests (*p* > 0.05), which is a natural distribution of the data in the real world (Figure 3A). Table 2 shows the mean and standard deviation of each LMC and other clinical information in detail. Except for NLA-SPT and ULT-ULL, all other LMC pairs had significant correlations (*p* < 0.05), confirming that they are closely related and influence each other (Figure 3B). The BMI was not normally distributed according to Kolmogorov-Smirnov tests (*p* < 0.05), with a mean of 19.41 ± 2.93 kg/m^2^ and mediation of 19.03 (17.58–20.08) (Figure 3C), leading to the speculation that this is due to increasing obesity. As for BMI categories, normal weight was the most prevalent with a percentage of 55.27%, underweight was the second most prevalent (40.93%), overweight was third (2.87%), and obese was the least frequent (0.93%) (Figure 3D). 

### 3.2. Associations between BMI and Lip Characteristics

The univariate analysis between BMI and LMCs indicated that NLA was negatively correlated with BMI, and the other LMCs were positively correlated with BMI (Figure 3E). However, this analysis did not consider confounders including demographic information, dental features, cephalometric indices on skeletal and incisor parameters and LMCs. To explore whether BMI independently affected LMCs, multivariable linear regression models were used, and the results are reported in Table 3. The result show that NLA was negatively correlated with BMI (model 1: β= −0.260, 95% CI −0.465 to −0.056; model 2: β= −0.342, 95% CI −0.529 to −0.154; model 3: β= −0.177, 95% CI −0.337 to −0.017), but not significantly after adjusting all considered covariates in model 4. On the contrary, SSD was positively correlated with BMI (model 1: β = 0.059, 95% CI 0.016 to 0.102; model 2: β= 0.044, 95% CI 0.007 to 0.080), but not significantly after adjusting LMCs or all covariates (model 3 and model 4). The same trend as for SSD can be seen in BULT and ULT, indicating that BMI did not independently influence these, but rather via other routes. The remaining four LMCs, ULL, SPT, ISD and LLL, had a significant positive correlation with BMI in all four models, suggesting that BMI is an independent factor influencing these four LMCs after adjusting for confounders.

### 3.3. Tendency of Lip Characteristics with BMI Variation

**Figure 4:**
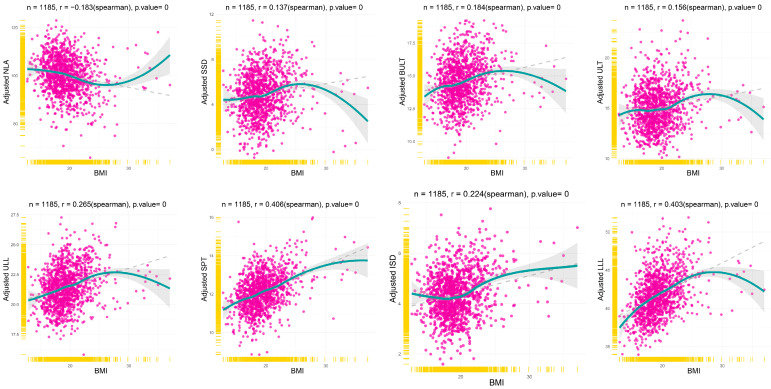
The relationships between the adjusted eight lip characteristics and BMI. The solid blue line fitted by the Loess function reveals the true variation tendency of lip characteristics according to the BMI, and the grey dashed lines fitted by the lm function reveal the general trend. NLA: Nasolabial angle, SSD: Superior sulcus depth, BULT: Basic upper lip thickness, ULT: Upper lip thickness, ULL: Upper lip length, SPT: Soft pogonion thickness, ISD: Inferior sulcus depth, LLL: Lower lip length, BMI: Body mass index.

**Figure 5:**
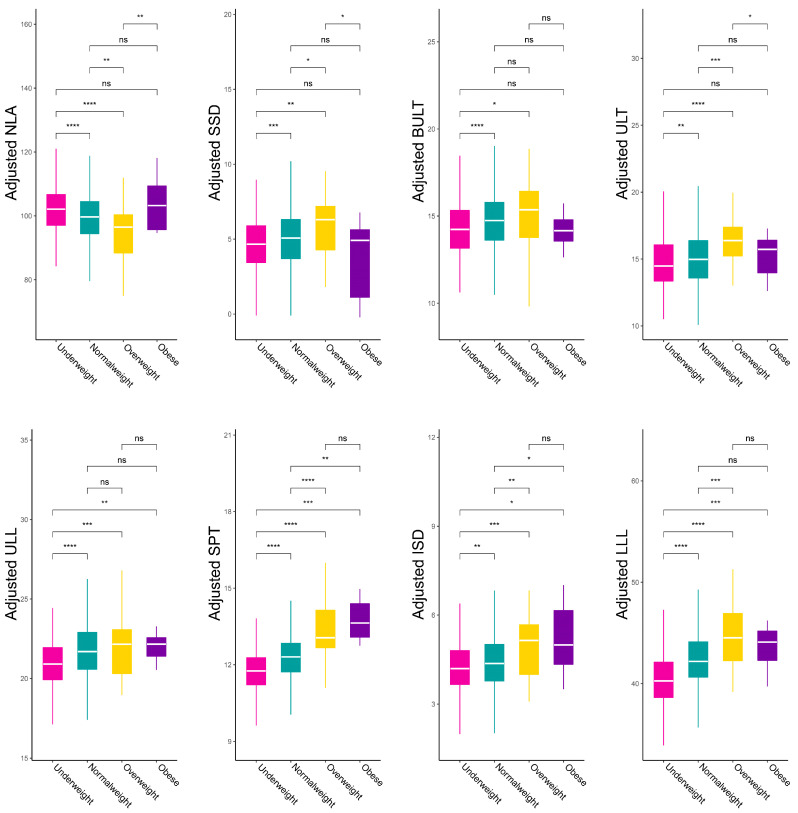
The comparations of multivariate regression analysis adjusted for eight lip characteristics among four BMI categories. NLA: Nasolabial angle, SSD: Superior sulcus depth, BULT: Basic upper lip thickness, ULT: Upper lip thickness, ULL: Upper lip length, SPT: Soft pogonion thickness, ISD: Inferior sulcus depth, LLL: Lower lip length, BMI: Body mass index. ns: *p* >= 0.05, *: *p* < 0.05, **: *p* < 0.01, ***: *p* < 0.001, ****: *p* < 0.0001.

To fully understand how LMCs vary with BMI, the scatter plot of the correlation between LMCs and BMI was made after adjusting for all covariates by multivariable linear regression (Figure 4). The local polynomial regression fitting of the blue line revealed the true variation tendency of LMCs according to the BMI. The gray dashed line was fitted by linear regression, revealing the general trend. The result shows that NLA was negatively correlated with BMI, while the other LMCs were positively correlated with BMI, which is in accordance with univariable analysis. Interestingly, the linear relationship did not hold in the obese category, which seemed to weaken or reverse the association. Furthermore, sensitivity analysis of BMI categories demonstrated similar results (Figure 5). Overweight had the smallest value of NLA and the biggest value for SSD, BULT, ULT, ULL, ISD and LLL. Only the SPT was gradually increased by BMI categories. These results suggest that obese patients have a different pattern of effects on LMCs, and the linear relationship between BMI and LMCs may only remain in non-obese patients. 

### 3.4. Mediation Analysis

**Figure 6:**
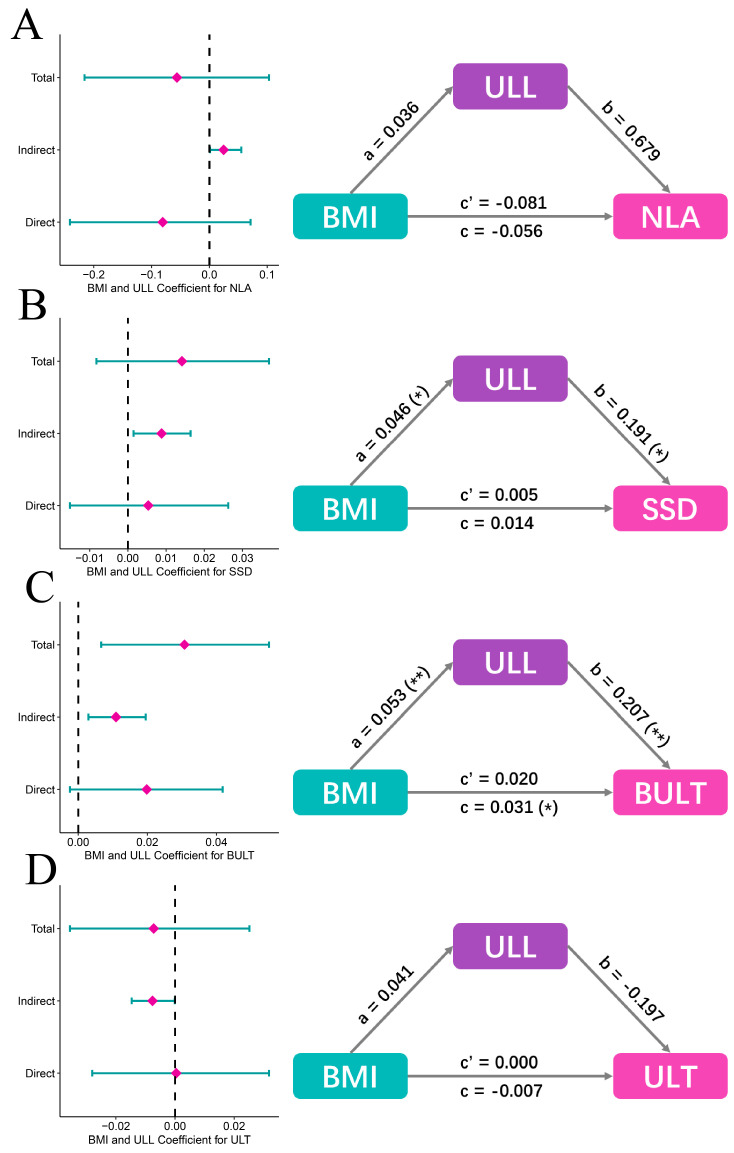
Mediation analysis among the four un-independent upper lip characteristics, ULL and BMI. (**A**) Mediation analysis model results of BMI, ULL and NLA (adjusted). Total effect (−0.056, *p* > 0.05) of BMI towards NLA consisted of direct effect (−0.081, *p* > 0.05) and indirect effect (0.025 = 0.036 × 0.679, *p* > 0.05). (**B**) Mediation analysis model results of BMI, ULL and SSD (adjusted). Total effect (0.014, *p* > 0.05) of BMI towards SSD consisted of direct effect (0.005, *p* > 0.05) and indirect effect (0.009 = 0.046 × 0.191, *p* = 0.018). (**C**) Mediation analysis model results of BMI, ULL and BULT (adjusted). Total effect (0.031, *p* = 0.014) of BMI towards BULT consisted of direct effect (0.020, *p* > 0.05) and indirect effect (0.011 = 0.053 × 0.207, *p* = 0.010). (**D**) Mediation analysis model results of BMI, ULL and ULT (adjusted). Total effect (−0.007, *p* > 0.05) of BMI towards ULT consisted of direct effect (0.000, *p* > 0.05) and indirect effect (−0.008 = 0.041 × (−0.197), *p* > 0.05). NLA: Nasolabial angle, SSD: Superior sulcus depth, BULT: Basic upper lip thickness, ULT: Upper lip thickness, ULL: Upper lip length, BMI: Body mass index. *: *p* < 0.05, **: *p* < 0.01.

Mediation analyses were performed to investigate why and how BMI related to NLA, SSD, BULT and ULT, while BMI is not an independent factor. The result shows that total effects (0.014, *p* > 0.05; 0.031, *p* = 0.014; respectively) of BMI toward SSD and BULT consisted of direct effect (0.005, 0.020 respectively, *p* > 0.05) and indirect effect (0.009 = 0.046 × 0.191, *p* = 0.018; 0.011 = 0.053 × 0.207, *p* = 0.010; respectively), indicating that BMI may relate to SSD and BULT through ULL (Figure 6B,C). However, total effect (−0.056, −0.007, *p* > 0.05, respectively) of BMI towards NLA and ULT consisted of direct effect (−0.081, 0.000, *p* > 0.05, respectively) and indirect effect (0.025 = 0.036 × 0.679, −0.008 = 0.041 × (−0.197), *p* > 0.05, respectively), indicating that BMI did not directly relate to NLA and ULT, or through ULL (Figure 6A,D). ULL is the only upper lip characteristic independently affected by BMI, thus we consider it a mediator.

## 4. Discussion

As the main part of the lower facial soft tissue, lips are vital to the perception of facial aesthetics. One of the primary concerns in orthodontic treatment is a coordinated and beautiful soft tissue profile, in which lips make a major contribution. Body Mass Index (BMI), the most widely used clinical measure of general obesity, has been recently found to affect facial bone and soft tissue structures [15]. Previous studies mainly focused on its relationship with FSTT and considered BMI as one of the key factors affecting FSTT. These findings are mainly applied to the field of forensic science for more detailed facial soft tissue reconstruction and facial recognition [24]. However, a recent study reported an association between BMI with linear lip measurements, but did not examine this in detail [22]. Lips being one of the most important factors affecting facial aesthetics, relevant studies on lip morphology characteristics and their association with BMI are few, and the relationship between the two is unclear [18,19]. Therefore, the study aimed to investigate the relationships between BMI and LMCs.

Most existing cephalometric analyses have been derived from orthodontics in Western countries, thus the reference values of cephalometric measurements were mainly standardized according to Caucasians [25,26]. However, there are significant differences in soft tissue characteristics and aesthetic preferences among different ethnic groups [1] and it is necessary to conduct a study with sufficient sample size in a Chinese population to facilitate the diagnosis and treatment planning of orthodontists and contribute to the development of facial reconstruction in the field of forensic science. In our study, the prevalence of different BMI categories and mean values of lip morphology characteristics, including NLA, SSD, BULT, ULT, ULL, SPT, ISD and LLL, were revealed in the Chinese population.

Previous studies have shown that facial soft tissue thickness (FSTT) increased with increase in BMI and that larger BMI was associated with increased linear measurements [18,22]. Similarly, we also demonstrated significant differences in lip characteristics across BMI categories and found that lip characteristics were positively correlated with BMI, except for nasolabial angle, which was negatively correlated with BMI. With increasing BMI, facial soft tissue thickness increases and, conceivably, the lip length and thickness measurements also increase, while the nasolabial angle decreases due to the protrusion of the upper lip, as confirmed by previous studies [7]. However, it is interesting that this linear relationship did not hold in obese patients. One possibility is that the sample size of the obese group was too small to detect a true trend. Another reason may be that the effect of increased BMI on soft tissue is limited. A very large BMI can cover all other anatomical factors [27]. In the obese category, changes in soft tissue size have reached their limits. This is similar to previous studies’ speculation that directional stretching of soft tissues is limited and there is mutual compensation [22,28]. Therefore, when BMI increases to a certain level, the increase in soft tissue volume may no longer influence length and thickness and will be compensated by width. Further research using three-dimensional imaging technology is needed to confirm this, as this study only involved cephalometric analysis and did not measure lip width. In addition, we suspected this, because the lip is closely related to the supporting hard tissue structures and is limited by adjacent structures [29], with the upper lip limited by the nose and the lower lip, and the lower lip limited by the upper lip and the chin, thus limiting its size variation in three dimensions. These results indicated that the BMI of patients should be considered in future treatment planning, and individualized treatment planning should be made because different soft tissue characteristics vary among different BMI. 

In addition, the independent association of LMCs with BMI was assessed in this study, considering various confounders. Multiple variables affecting soft tissue morphology have been identified. Many studies have focused on age, with several studies showing that the upper and lower lips significantly retruded compared to the aesthetic line and became thinner with aging [30,31,32]. Gender is also an important factor in soft tissue morphology, and it has been demonstrated in many different countries and regions that facial soft tissue including the lips of males is thicker than that of females [30,33,34]. Besides, race-related soft tissue differences have also been commonly involved [35,36,37]. Basically, the Negroid population have the thickest and the most protruding lips, followed by the Asian population, with the Caucasian population the thinnest and straightest, and with shorter upper lips. In addition, the incisor position and skeletal patterns are also confounding factors highlighted by numerous studies [6,7,38], which show that upper lip thickness and length are significantly correlated with the protrusion of maxillary incisors, and there are significant differences in the upper lip morphology among different skeletal patterns. After adjusting for confounding variables that may affect the relationship between lip characteristics and BMI, ULL, SPT, ISD and LLL were revealed to be positively correlated with BMI. Previous studies simply explored the relationship between soft tissue thickness and BMI, and concluded that obese subjects have thicker gnathion and pogonion soft tissue thickness. However, these studies did not include sufficient sample size, adequately adjust for confounding factors, or specifically measure and compare lip characteristics among different BMI categories [19,39,40]. In clinical practice, teenage patients, who account for a large proportion of orthodontic treatments, are at the peak of their growth and development, with rapid increases in height and weight and changes in BMI. The results of this study can help doctors predict the possible impact of BMI changes on soft tissue profile in advance. Besides, BMI has been shown to influence skeletal development, with obese/overweight children and adolescents more likely to experience advanced dental and skeletal maturation [41,42,43], thus influencing the timing of intervention and treatment plans. 

For adult patients, studies have shown that the mandibular cortex was thicker in obese and overweight patients, and periodontal tissue responded differently to orthodontic force [41,44]. Moreover, it has been found that increased BMI may be related to the decrease of orthodontic treatment compliance, which is worthy of attention [14]. However, it was found that NLA, SSD, BULT and ULT were not independently affected by BMI, and mediation analysis found BMI associated with SSD and BULT through ULL, which is an unprecedented discovery. This may indicate that changes in the length of the upper lip are limited by the adjacent structures, so when the length of the upper lip increases to a certain extent, it is compensated for by changes in other dimensions, such as the basic upper lip thickness, which in turn affects the depth of the upper lip groove. However, the result of mediation analysis between the other two upper lip morphology characteristics, NLA and ULT, with BMI, was not significant. We speculate that the sample size is insufficient to find statistical differences or that there are lip characteristics which have not been measured, and further research could be considered to focus on this. 

To the best of our knowledge, the current study is the first to explore the association of BMI with lip morphology characteristics in a Chinese population, and the non-negligible influence of BMI on lip morphology has been found, which provides a further reference for the diagnosis, personalized treatment planning and subsequent scientific research in orthodontics. Based on previous studies and the large sample size of this study, multiple linear regression was used to adequately adjust for covariates, thus increasing the accuracy and authenticity of the results [45,46]. Furthermore, it should not be ignored that special mediation analysis was used to further explore the lip characteristics not independently affected by BMI, and the mediator ULL was found.

The study has several limitations. First, as a cross-sectional study, we could only draw conclusions on whether there was a correlation between BMI and LMCs, rather than a direct causal relationship. Second, due to limitations of the database, we were unable to identify participants younger than 12 y, older than 53 y, with a history of orthodontic treatment or without permanent dentition, hence the conclusions need to be interpreted with caution. Third, covariates were adjusted to control for confounders, but there may still be unmeasured or unknown covariates, such as waistline, body fat percentage and systemic diseases such as diabetes and hypertension, etc. Finally, the sample source of this study is a Chinese population, so the findings should be cautiously generalized to other populations. Future studies can use frontal photographs and three-dimensional facial scanning techniques to focus on more lip features from different perspectives and explore their relationship between BMI. More diverse groups such as children and the elderly should also be considered.

## 5. Conclusions

1. The study provided the reference values of lip morphology characteristics and BMI in a Chinese population, which can provide a reference for Chinese doctors and researchers.
2. After adjusting for confounders, lip morphology characteristics were found to be significantly different among various BMI categories, suggesting that orthodontists should develop more personalized treatment options for patients with different BMI.
3. BMI is associated with all LMCs, but only independently associated with upper lip length, soft pogonion thickness, inferior sulcus depth and lower lip length.
4. The relationship between BMI and LMCs in obese patients was different from that in other BMI categories.
5. BMI was associated with superior sulcus depth (SSD) and basic upper lip thickness (BULT) through upper lip length (ULL).

## Figures and Tables

**Table 1:** Cephalometric landmarks used in this study.

Landmarks	Definition
Porion (Po)	The most superior point of the anatomical image of the external auditory canal.
Orbitale (Or)	The lowest point of the inferior orbital margin.
Sub-spinale (point A)	The most concave point of the bone between anterior nasal spine and upper alveolar margin.
Pogonion (Pog)	The most protruding point of the chin.
Columella (Cm)	The most prominent point below the nasal contour at the junction with the nasal tip.
Sub-nasale (Sn)	The junction of the nasal columella with the upper lip.
Labrale superius (UL)	The most prominent point on the upper lip.
Stomion superius (Stms)	The lowest point of the upper lip vermilion margin.
Stomion inferius (Stmi)	The highest point of the lower lip vermilion margin.
Labrale inferius (LL)	The most prominent point on the lower lip.
Mento-labial sulcus (Si)	The most concave point between the chin and lower lip.
Pogonion of soft tissue (Pog’)	The most anterior point of soft tissue of the chin.
Menton of soft tissue (Me’)	The lowest point of soft tissue of the chin.

**Table 2:** Demographic and clinical characteristics of participants stratified by BMI category.

Level	Overall	Underweight	Normal Weight	Overweight	Obese	*p*-Value
N	1185	485	655	34	11	
Demographic characteristics						
BMI (median (IQR))	19.03 (17.58, 20.82)	17.19 (16.23, 17.88)	20.29 (19.25, 21.72)	26.26 (25.59, 27.61)	32.87 (32.55, 34.14)	<0.001
Age (median (IQR))	19 (14, 24)	15 (13. 21)	20 (16, 25)	18 (12, 22)	15 (13, 21)	<0.001
Gender (%)						
Male	396 (33.4)	136 (28.0)	234 (35.7)	21 (61.8)	5 (45.5)	<0.001
Female	789 (66.6)	349 (72.0)	421 (64.3)	13 (38.2)	6 (54.5)	
Dental characteristics						
Molar Relationship (%)						
I	416 (35.1)	174 (35.9)	226 (34.5)	12 (35.3)	4 (36.4)	0.034
II-1	381 (32.2)	175 (36.1)	196 (29.9)	7 (20.6)	3 (27.3)	
II-2	111 (9.4)	49 (10.1)	59 (9.0)	2 (5.9)	1 (9.1)	
III	277 (23.4)	87 (17.9)	174 (26.6)	13 (38.2)	3 (27.3)	
Upper crowding (%)						
I	685 (57.8)	263 (54.2)	393 (60.0)	22 (64.7)	7 (63.6)	0.24
II	319 (26.9)	141 (29.1)	171 (26.1)	6 (17.6)	1 (9.1)	
III	181 (15.3)	81 (16.7)	91 (13.9)	6 (17.6)	3 (27.3)	
Lower crowding (%)						
I	740 (62.4)	291 (60.0)	415 (63.4)	26 (76.5)	8 (72.7)	0.309
II	336 (28.4)	149 (30.7)	180 (27.5)	6 (17.6)	1 (9.1)	
III	109 (9.2)	45 (9.3)	60 (9.2)	2 (5.9)	2 (18.2)	
Overbite (mm) (median (IQR))	2.50 (1.00, 4.10)	2.80 (1.10, 4.10)	2.40 (0.90, 4.10)	2.10 (0.35, 4.25)	4.00 (2.20, 5.25)	0.065
Overjet (mm) (median (IQR))	4.0 (2.5, 5.7)	4.3 (2.7, 5.8)	3.7 (2.4, 5.7)	3.0 (−0.8, 5.3)	5.2 (2.9, 6.5)	0.037
Skeletal characteristics						
SNA (mean (SD))	80.82 (3.69)	80.58 (3.42)	80.90 (3.86)	82.08 (3.72)	82.74 (3.44)	0.025
SNB (mean (SD))	77.42 (4.51)	76.85 (4.07)	77.72 (4.73)	79.36 (5.44)	78.40 (3.31)	0.001
ANB (median (IQR))	3.9 (1.6, 5.6)	4.1 (2.0, 5.8)	3.6 (1.3, 5.5)	4.0 (−0.2, 5.8)	4.1 (2.4, 6.3)	0.070
SN-MP (mean (SD))	34.50 (7.04)	35.12 (6.59)	34.22 (7.22)	32.00 (8.94)	31.85 (6.59)	0.014
FH-MP (mean (SD))	24.60 (6.56)	25.15 (6.44)	24.26 (6.55)	23.54 (8.20)	23.85 (6.82)	0.104
U1-NA (mm) (mean (SD))	5.56 (2.84)	5.36 (2.82)	5.74 (2.83)	5.86 (3.20)	3.56 (2.37)	0.013
U1-SN (median (IQR))	109 (103, 115)	109 (103, 115)	110 (102, 115)	110 (106, 120)	107 (100, 112)	0.20
L1-NB (mm) (mean (SD))	6.43 (3.00)	6.25 (2.80)	6.58 (3.13)	6.59 (3.41)	5.06 (2.47)	0.13
L1-MP (mean (SD))	96.42 (9.38)	96.16 (8.97)	96.58 (9.68)	98.04 (8.37)	94.04 (11.79)	0.517
Lip morphology characteristics						
Nasolabial angle (NLA) (mean (SD))	100.25 (10.41)	101.74 (10.44)	99.34 (10.09)	95.45 (11.79)	103.33 (13.91)	<0.001
Superior sulcus depth (SSD) (mm) (mean (SD))	4.88 (2.18)	4.62 (2.02)	5.01 (2.25)	6.16 (2.40)	3.80 (2.52)	<0.001
Basic upper lip thickness (BULT) (mm) (mean (SD))	14.55 (2.00)	14.24 (1.86)	14.75 (2.05)	15.24 (2.60)	14.46 (1.51)	<0.001
Upper lip thickness (ULT) (mm) (mean (SD))	15.01 (2.52)	14.76 (2.45)	15.12 (2.56)	16.40 (2.56)	14.56 (1.86)	0.001
Upper lip length (ULL) (mm) (mean (SD))	21.46 (2.33)	21.00 (2.09)	21.77 (2.41)	22.09 (2.91)	21.42 (1.77)	<0.001
Soft pogonion thickness (SPT) (mm) (mean (SD))	12.12 (2.36)	11.66 (2.24)	12.42 (2.34)	13.44 (2.56)	10.42 (2.99)	<0.001
Inferior sulcus depth (ISD) (mm) (mean (SD))	4.36 (1.35)	4.19 (1.29)	4.46 (1.37)	4.78 (1.59)	4.68 (1.43)	0.001
Lower lip length (LLL) (mm) (mean (SD))	41.75 (4.26)	40.29 (4.04)	42.69 (4.05)	44.60 (5.16)	41.02 (3.17)	<0.001

**Table 3:** Association of BMI with upper and lower lip characteristics by multivariate analysis.

	β (95% CI)			
	Model 1 ^a^	Model 2 ^b^	Model 3 ^c^	Model 4 ^d^
Nasolabial angle (NLA)	−0.260 * (−0.465, −0.056)	−0.342 *** (−0.529, −0.154)	−0.177 * (−0.337, −0.017)	−0.081 (−0.235, 0.074)
Superior sulcus depth (SSD) (mm)	0.059 ** (0.016, 0.102)	0.044 * (0.007, 0.080)	−0.002 (−0.035, 0.030)	0.005 (−0.017, 0.027)
Basic upper lip thickness (BULT) (mm)	0.070 *** (0.033, 0.107)	0.092 *** (0.055, 0.129)	0.013 (−0.021, 0.048)	0.020 (−0.004, 0.044)
Upper lip thickness (ULT) (mm)	0.098 *** (0.053, 0.142)	0.090 *** (0.045, 0.135)	−0.002 (−0.047, 0.043)	0.0004 (−0.031, 0.032)
Upper lip length (ULL) (mm)	0.084 *** (0.042, 0.126)	0.147 *** (0.108, 0.186)	0.060 ** (0.018, 0.103)	0.039 * (0.002, 0.075)
Soft pogonion thickness (SPT) (mm)	0.147 *** (0.100, 0.194)	0.155 *** (0.111, 0.200)	0.091 *** (0.043, 0.139)	0.120 *** (0.073, 0.168)
Inferior sulcus depth (ISD) (mm)	0.076 *** (0.053, 0.099)	0.074 *** (0.051, 0.097)	0.070 *** (0.045, 0.096)	0.040 *** (0.018, 0.063)
Lower lip length (LLL) (mm)	0.269 *** (0.193, 0.344)	0.352 *** (0.283, 0.422)	0.278 *** (0.206, 0.351)	0.208 *** (0.139, 0.276)

Note: * *p* < 0.05; ** *p* < 0.01; *** *p* < 0.001. Abbreviation: β, coefficient of BMI in multivariate analysis; CI, confidence interval. ^a^ Model 1 was adjusted for “Age”, “Gender”, “Molar Relationship”, “Upper crowding”, “Lower crowding”, “Overbite” and “Overjet”. ^b^ Model 2 was adjusted for “SNA”, “SNB”, “ANB”, “SN-MP”, “FH-MP”, “U1-NA (mm)”, “U1-SN” “L1-NB (mm)” and “L1-MP”. ^c^ Model 3 was adjusted for “Nasolabial angle (NLA)”, “Superior sulcus depth (SSD) (mm)”, “Basic upper lip thickness (BULT) (mm)” “Upper lip thickness (ULT) (mm)”, “Upper lip length (ULL) (mm)”, “Soft pogonion thickness (SPT) (mm)”, “Inferior sulcus depth (ISD) (mm)” and “Lower lip length (LLL) (mm)”. ^d^ Model 4 was adjusted for all above variables.

## Data Availability

Not applicable.
